# Preparation of uniform-sized GeXIVA[1,2]-loaded PLGA microspheres as long-effective release system with high encapsulation efficiency

**DOI:** 10.1080/10717544.2022.2089297

**Published:** 2022-07-22

**Authors:** Lu Li, Zhiguo Li, Yongxin Guo, Kai Zhang, Weidong Mi, Jing Liu

**Affiliations:** a Heilongjiang University of Traditional Chinese medicine, Harbin, China; b Institute of Pharmacology and Toxicology, Academy of Military Medical Sciences, Beijing, China; c Department of Anesthesiology, The First Medical Center of the PLA General Hospital, Beijing, China

**Keywords:** α-Conotoxin GeXIVA[1,2], PLGA microspheres, narrow size distribution, encapsulation efficiency, molecular simulation

## Abstract

The purpose of this study was to prepare GeXIVA[1,2] PLGA microspheres by W/O/W re-emulsification-solvent evaporation technology and to develop sustained-release formulations to meet the clinical treatment needs of chronic neuropathic pain. Through prescription optimization, the uniformity of particle size and the encapsulation efficiency is improved, so as to achieve the quality standard of the microspheres. The mechanism of trehalose improving the stability of GeXIVA[1,2] was studied and verified by molecular simulation. The results showed that when adding trehalose to W1, using the PLGA model of 75:25, PLGA concentration of 30%, PVA concentration of 1.5%, adding 1% NaCl to PVA and adding 1% NaCl to solidification water, the prepared microspheres are smooth, the particle size is about 25 μm, and the encapsulation rate reaches 90%. The results of in vitro release experiments showed that the microspheres could be released steadily for about 30 days. The microsphere samples were characterized and analyzed by molecular simulation and powder X-ray diffractometer, and the protective mechanism of trehalose on GeXIVA[1,2] was discussed. The results showed that the hydrogen bond formed between trehalose and GeXIVA[1,2] acted as a hydration film and played a certain protective role on GeXIVA[1,2]. In addition, high-viscosity trehalose can form a glass state and wrap around GeXIVA[1,2], reducing the free movement of molecules. In the microsphere system, trehalose can also avoid the influence of PLGA material on the secondary structure of GeXIVA[1,2]. In conclusion, this study is expected to provide a new therapeutic strategy for the treatment of chronic neuropathic pain.

## Graphical Abstract

**Figure:**
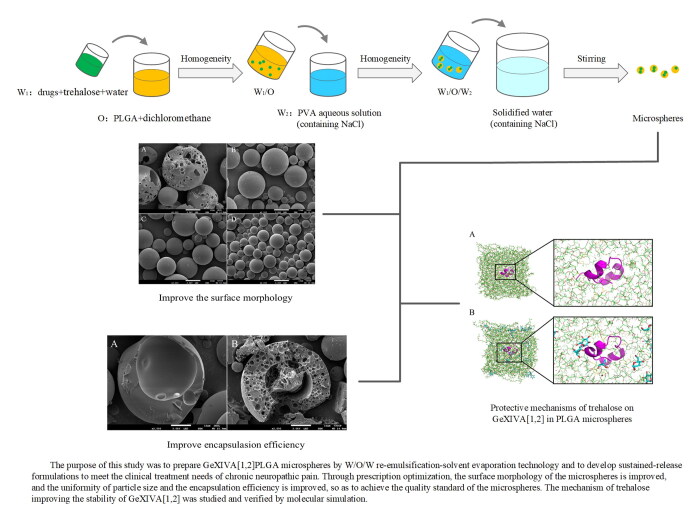

## 1. Introduction

Currently, the drug of choice for the clinical treatment of chronic neuralgia is opioids. However, opioids are accompanied by a series of adverse reactions such as tolerance and addiction, and they are frequently administered, resulting in poor patient compliance, and there are certain limitations in clinical treatment (Angst & Clark, 2006; Chou et al., 2015; Devereaux et al., 2018). Conotoxin αO-GeXIVA, a 28-amino acid polypeptide found in the marine animal conus in the South country Sea, can achieve an analgesic effect by specifically blocking the α9α10 nAChR receptor, which has attracted widespread attention (Yu et al., 2018; Xu et al., 2020; Wang et al., 2019). Its isomer GeXIVA[1,2] is by far the most active conotoxin (Yousuf et al., 2022), showing excellent analgesic effects in many disease models (Vincler & Mcintosh, 2007; Alsharari et al., 2020; Khan et al., 2002). Notably, no addictive or motor side effects were observed during the treatment period. Therefore, GeXIVA[1,2], as a novel polypeptide compound with unique pharmacological and analgesic effects, is expected to be used in the clinic to improve the therapeutic effect of chronic neuropathic pain.

The microsphere drug delivery system has a long-acting and sustained-release effect, which can release the encapsulated drugs for several weeks to several months (Jeyanthi et al., 1997). At present, it has been successfully applied to a variety of polypeptide drugs and has become an important direction for the research and development of sustained and controlled release preparations (Shive & Anderson, 1997, Yang et al., 2000). Therefore, the development of GeXIVA[1,2] as a microsphere sustained-release preparation has great advantages in the treatment of chronic neuropathic pain, such as reducing the frequency of dosing, thereby enhancing patient compliance; The system administration of degradable microspheres can prevent the rapid degradation of the drug in the body, increase the stability of the drug, and reduce the fluctuation of the blood drug concentration (Diwan & Park, 2001).

The preparation methods of sustained-release microspheres mainly include the emulsification-solvent evaporation method (Mcginity & O’donnell, 1997, Latha et al., 2021), spray drying method (Giunchedi et al., 2001, Wang et al., 2008), phase separation method (Fu et al., 2012), membrane emulsification method (Wang et al., 2005), microfluidic method (Wang et al., 2018), and hot-melt extrusion method (Guo et al., 2017). The emulsification-solvent evaporation method is the most widely used preparation method in the marketed microsphere products. For water-soluble drugs such as polypeptides, the W/O/W re-emulsification-solvent evaporation method is the preferred preparation method (Uchida et al., 1996). The W/O/W double emulsification method to prepare microspheres is characterized by a simple preparation process, good process reproducibility, and controllable particle size range of microspheres.

It should be noted that many factors that affect the quality of microsphere products (Hua et al., 2021), and the uniformity of particle size of microspheres is a key factor that significantly affects the performance of microspheres (Qi et al., 2013). Small particle size microspheres with high specific surface area will lead to higher burst release or faster release rate in vitro (Duncan et al., 2005, Klose et al., 2006), which is not suitable for long-term release to achieve sustained analgesic effect. Therefore, it is very necessary and important to prepare microspheres with uniform particle size. On the other hand, during the preparation of microspheres, the stability of the peptide drug will decrease, resulting in loss, resulting in a decrease in the encapsulation efficiency, which is mainly caused by two factors: one is the emulsification and homogenization during the preparation of primary emulsion (W_1_/O). The resulting shear stress produces mechanical damage; another is due to the diffusion of the concentration gradient, the drug in the inner aqueous phase leaks into the outer aqueous phase due to its better solubility, resulting in the loss of the drug (Jain et al., 2000). Using the emulsification-solvent evaporation method to prepare polypeptide microspheres, mechanical damage is unavoidable in the process, and the diffusion of polypeptides can be adjusted by changing the formulation variables, for example, adjusting the volume of the internal water phase, changing the emulsification method, adding excipients, Adjust the osmotic pressure, change the curing rate of the polymer, etc. (Zhu et al., 2001, Park et al., 2019). Adjusting these factors may directly or indirectly affect the loss of polypeptides, which in turn affects the encapsulation efficiency of polypeptide microspheres.

The study aimed to demonstrate how useful optimization of the prescription during the preparation phase is for improving the final product quality of drug-loaded PLGA microsphere formulations. In our present study, we mainly studied the effects of various prescription factors such as the concentration of sodium chloride in solidified water, the ratio of GA-LA to PLGA, and the concentration of PLGA on the surface morphology and entrapment efficiency of GeXIVA[1,2] microspheres were investigated. The microsphere samples were characterized by differential scanning calorimetry (DSC), powder X-ray diffractometer (PXRD) and Fourier transform infrared spectroscopy (FTIR). Molecular simulations were also used to understand the internal structure and interactions between trehalose and GeXIVA [1,2] and PLGA.

## 2. Materials and methods

### 2.1. Materials

GeXIVA[1,2] API was provided by Gill Biochemical Co., Ltd. (Shanghai). PLGA 75/25 was purchased from EVONIK. Trehalose was injection-grade and was purchased from Hayashihara Co., Ltd. (Okayama, Japan). Polyvinyl alcohol (PVA) was purchased from Kuraray co., ltd. HPLC-grade triethylamine and acetonitrile were obtained from Thermo Fisher Scientific (Waltham, MA, USA). The purified water used in this study was prepared using a Mille-Q system (EMD Millipore, Billerica, MA, USA). All other reagents were of analytical grade and were purchased from Sinopharm Chemical Reagent Co., Ltd.

### 2.2. Preparation of GeXIVA[1,2]-loaded PLGA microspheres

Various formulations of GeXIVA[1,2]-loaded PLGA microspheres were prepared by the double emulsion (W/O/W)-solvent evaporation method. Briefly, 20 mg of GeXIVA[1,2] was dissolved in 0.1 mL of distilled water with or without additives as inner water phase (W_1_). Aqueous GeXIVA[1,2] solution was mixed with 2.5 mL dichloromethane (DCM) containing PLGA, and then emulsified using a homogenizer (T-18 Basic ULTRA-TURRAX®, IKA^®^-WERKE GMBH & Co. KG, Germany) at 19,000 rpm for 120 s in ice bath. This primary emulsion (W_1_/O) was then added to 25 mL of outer water phase (W_2_) containing 1% poly vinyl alcohol (PVA) with or without NaCl. The emulsification continued at 8,000 rpm for 180 s to form the secondary emulsion (W_1_/O/W_2_). After that, the emulsion droplets were poured into 250 mL of distilled water containing NaCl or not, and then stirred at 250 rpm for 8 h at room temperature to remove DCM. The solidified microspheres were washed three times with distilled water by centrifugation at 4,000 rpm for 10 min (Eppendorf centrifuge 5019 R, Hamburg, Germany), and then freeze-dried. The samples were frozen at an initial shelf temperature of −45 °C for 6 h. Primary drying was conducted at a chamber pressure of 0.2 millibar with shelf temperature adjusted to −10 °C and held for 10 h. Following primary drying, shelf temperature was increased to 20 °C at a ramp rate of 10 °C/h and secondary drying was performed for about 6 h.

### 2.3. Particle size distribution measurement

Particle size and size distribution of GeXIVA[1,2]-loaded PLGA microspheres was measured by Mastersizer 2000 (Malvern Instrument, Malvern, UK). Particle sizes of microspheres are expressed as the volume mean diameter (VMD) in micrometers (μm). The particle size distribution was referred as Span value and calculated as follows:

$$\mathrm{Span}=\frac{D90\%-D10\%}{D50\%}\;\times \;100\%$$

where D90%, D50% and D10% are VMD at 90%, 50% and 10% of the cumulative volume, respectively. The smaller Span value indicates the narrower size distribution.

### 2.4. Morphology observation

Scanning electron microscopy (SEM) was performed to observe the shape and morphology of the PLGA microspheres by using a JSM-7900F (JEOL, Hiroshima, Japan). Microspheres were placed on a metal sample stub using conductive carbon double-sided adhesive tape. Gold-palladium coating was applied using an automatic sputter coater. The morphology and appearance of samples were examined at an accelerating voltage of 3.0 kV.

### 2.5. Drug loading efficiency (LE) and encapsulation efficiency (EE)

A total of 20 mg of microspheres were dissolved in 7 mL of acetonitrile, to which 3 mL of distilled water was then added. The mixture was agitated to dissolve GeXIVA[1,2] completely. Next, the solution was filtered and then injected into a RP-HPLC system to determine the concentration of GeXIVA[1,2]. A RP-HPLC column (Kromasil-100, 3.5 μm × 4.6 mm × 250 mm) was used for the chromatographic separations. The mobile phase consisted of acetonitrile and 0.05% triethylamine in water (14:86, *v/v*). Samples were eluted in isocratic mode with a flow rate of 1.0 mL/min at 40 °C. The detection wavelength was set at 215 nm and the injection volume was 20 μL. Drug Loading efficiency (LE) and Encapsulation Efficiency (EE) of the microspheres were calculated by the following equations:

$$\mathrm{LE}(\%,w/w)=\frac{\text{Mass ofdruginmicrospheres}}{\text{Mass ofmicrospheres}}\;\times \;100\%$$

$$\mathrm{EE}(\%,w/w)=\frac{\text{Measured LE}}{\text{Theoretical LE}}\;\times \;100\%$$

### 2.6. In vitro release of GeXIVA[1,2]-loaded microspheres

Approximately 20 mg GeXIVA[1,2]-loaded PLGA microspheres were incubated in 5 mL of 10 mM phosphate buffer saline (PBS, pH7.4) containing 0.01% sodium azide under agitation (100 rpm) at 37 °C. At predetermined time intervals, supernatants were periodically collected by centrifugation at 4,000 rpm for 10 min and replaced with fresh buffer of equal volume. The concentration of GeXIVA[1,2] in the supernatant was determined by the RP-HPLC method described above. All the release experiments were done in triplicate.

### 2.7. Differential scanning calorimetry (DSC)

Differential scanning calorimetry (DSC) was performed in a DSC Q2000 (TA Instruments, New Castle, DE, USA) equipped with a TA instrument Universal Analysis software, an autosampler and a cooling system. Nitrogen gas was purged at a pressure of 20 psi to provide an inert atmosphere and prevent oxidation during measurement. About 5 mg of microspheres sample was placed in a crimped aluminum hermetic pan and DSC was performed at a ramp rate of 5 °C/min to 200 °C. Individual GeXIVA[1,2] and PLGA in pure non-lyophilized form were also scanned to obtain the thermograms for comparison.

### 2.8. Fourier transform infrared spectroscopy (FTIR)

Fourier transform infrared spectra (FTIR) were collected for samples using a Bruker Tensor 27 (Bruker Optics, Billerica, MA, USA) equipped with an attenuated total re-flection (ATR) accessory. All the samples were measured in the frequency range of 500–4000 cm^−1^ with a data density of 4 cm^−1^ and a total of 50 scans using OPUS software (Bruker Optics, version 7.0). Prior to the collection of spectra for the samples, background spectra were scanned first by purging the detector with nitrogen gas to minimize interference by water vapor and CO_2_.

### 2.9. Molecular Computations

The generation amber force field (GAFF) was used for trehalose and PLGA with restrained electro static potential (RESP) charge applied to these molecules. The leap.ff14SB.protein force filed was employed for GeXIVA[1,2]. We first performed energy minimization for 5000 steps, and then restrained the heavy atoms with a constant force of 1000 kJ/mol/nm^2^ for 100 ps, the final production simulations were performed for 200 ns for the systems. The simulation temperature and pressure were set to 300 K with v-rescale coupling method and 1 atm with bredensen coupling method. The hydrogen bond was restrained by the LINC algorithm, which allows us safely to set the time step to 2 fs. All the simulations were performed and the simulation results were analyzed by GROMACS 2018.

## 3. Results and discussion

### 3.1. Evaluation of GeXIVA[1,2]-loaded PLGA microspheres prepared in the basic formulation

**Figure 1.**
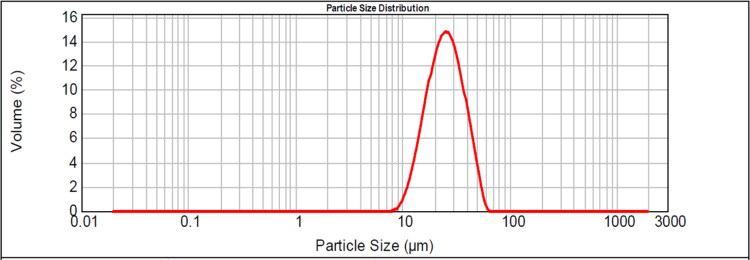
Particle size and distribution of GeXIVA[1,2] microspheres prepared by basic formula.

In the basic formulation, no additive was added in W_1_, and no NaCl was contained in W_2_ or curing water. PLGA 7525 was used in the oil phase at the concentration of 10%. Other operations were carried out as described above The proprieties of GeXIVA[1,2] microspheres prepared by the basic formulation were evaluated. It showed that the average particle size of the microspheres was 25.44 μm, which was also verified by the SEM results. The results meant the laser particle size analyzer had good accuracy in detecting the particle size of the microspheres. The Span value of the microspheres was 1.19, implying a quite narrow size distribution (Figure 1).

**Figure 2.**
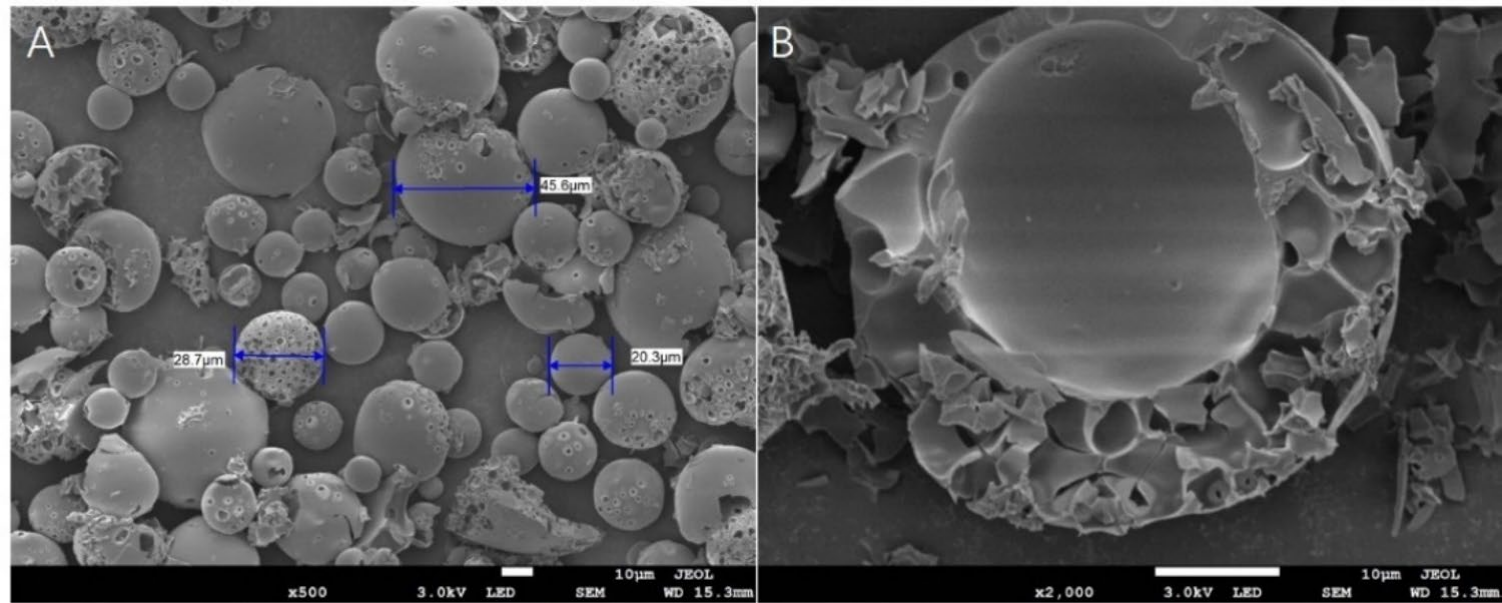
Scanning electron microscope image of GeXIVA[1,2] microspheres prepared by basic recipe: (A) Surface morphology of microspheres; (B) Internal structure of microspheres.

However, poor morphology was observed from the results of SEM (Figure 2). Most of the microspheres were in irregular shape, and large holes formed on the surface. Some microspheres were even broken. Imperfect structure and shape of the microspheres may lead to the leakage of GeXIVA[1,2] and eventually trigger the low LE and EE. As a result, the LE and EE were only 0.53% and 13.88%, respectively.

This strategy has, to some extent, proved effective in preparing GeXIVA[1,2]-loaded PLGA microspheres. However, the appearance and EE of microspheres need to be further improved to ensure the good qualities.

### 3.2. Optimization for preparing GeXIVA[1,2]-loaded PLGA microspheres with good morphology

#### 3.2.1. Concentration of NaCl in solidification water

**Figure 3.**
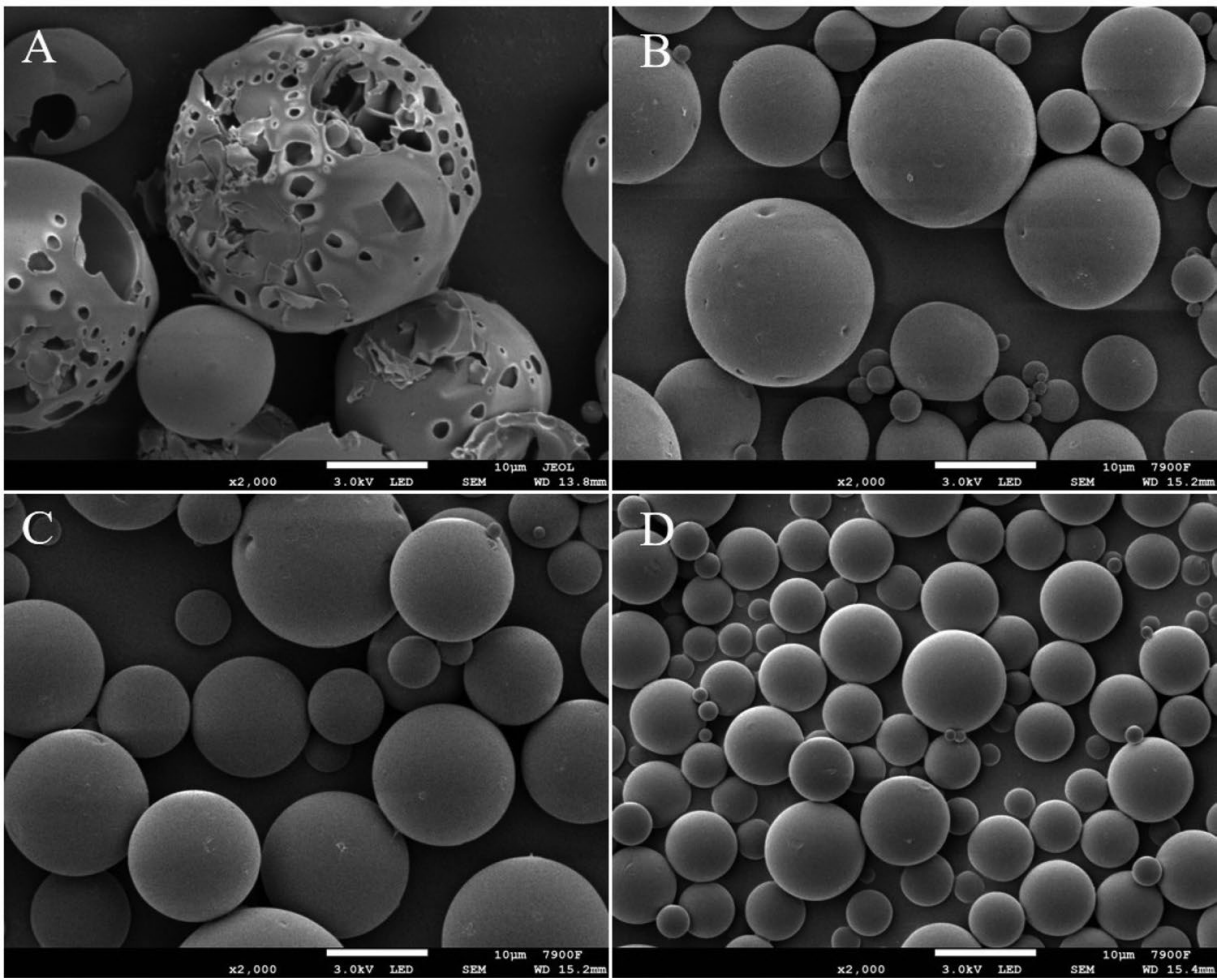
The effect of different NaCl concentration on the morphology of microspheres in solidified water: (A) 0%NaCl; (B) 0.5%NaCl; (C) 1.0%NaCl; (D) 2.0%NaCl.

The results showed that when the water for curing does not contain NaCl, the morphology of the microspheres was poor, and large holes were formed on the surface and inside of the microspheres, which will lead to the internal drug leakage directly. It would not only affect the morphology of the microspheres, but also have a great impact on the EE and LE. The morphology of the microspheres can be improved by adding NaCl to the water for solidification. The addition of NaCl has a certain effect on the particle size of the microspheres, but changing the concentration of NaCl has no obvious effect on the particle size and size distribution of the microspheres. The addition of NaCl had no significant effect on the encapsulation efficiency of the microspheres. In conclusion, when the concentration of NaCl is 1.0%, the morphology of the microspheres is better, and the encapsulation efficiency is the highest. Therefore, the concentration of NaCl added to the water for solidification is 1.0% (Table 1, Figure 3).

**Table 1.** The effect of NaCl concentration on the particle size and encapsulation efficiency of microspheres (*n* = 3).

NaCl concentration	Average particle size (μm)	Span	EE (%)
0%	25.44 ± 0.12	1.19	13.88 ± 0.13
0.5 %	16.56 ± 0.09	1.22	13.60 ± 0.32
1.0 %	16.01 ± 0.11	1.03	14.37 ± 0.16
2.0 %	14.86 ± 0.05	1.07	11.99 ± 0.19

#### 3.2.2. LA-GA ratio of PLGA

Table 2 showed that when the LA: GA ratio of PLGA was 85:15, 75:25 and 50:50, the particle size of PLGA microspheres gradually decreased to 29.48 μm, 16.01 μm and 10.53 μm, respectively, which was more obvious compared with the drug loading of PLGA microspheres. This phenomenon was consistent with the results of other studies. It is speculated that with the increase of GA ratio, the hydrophilicity of the polymer will also be improved, and the increase of hydrophilicity will reduce the surface tension of the droplets, and the smaller droplets will be formed under the action of shear force, and the final microspheres will be smaller. In order to obtain drug loaded microspheres with larger particle size, higher drug loading and longer release time, the La: GA ratio of 75:25 was selected.

**Table 2.** Effects of different types of PLGA on particle size and encapsulation efficiency.

LA/GA ratio of PLGA	Average particle size (μm)	Span	EE (%)
85:15	29.48 ± 0.03	0.85	10.56 ± 0.39
75:25	16.01 ± 0.11	1.03	14.37 ± 0.16
50:50	10.53 ± 0.12	1.58	13.88 ± 0.72

**Figure 4.**
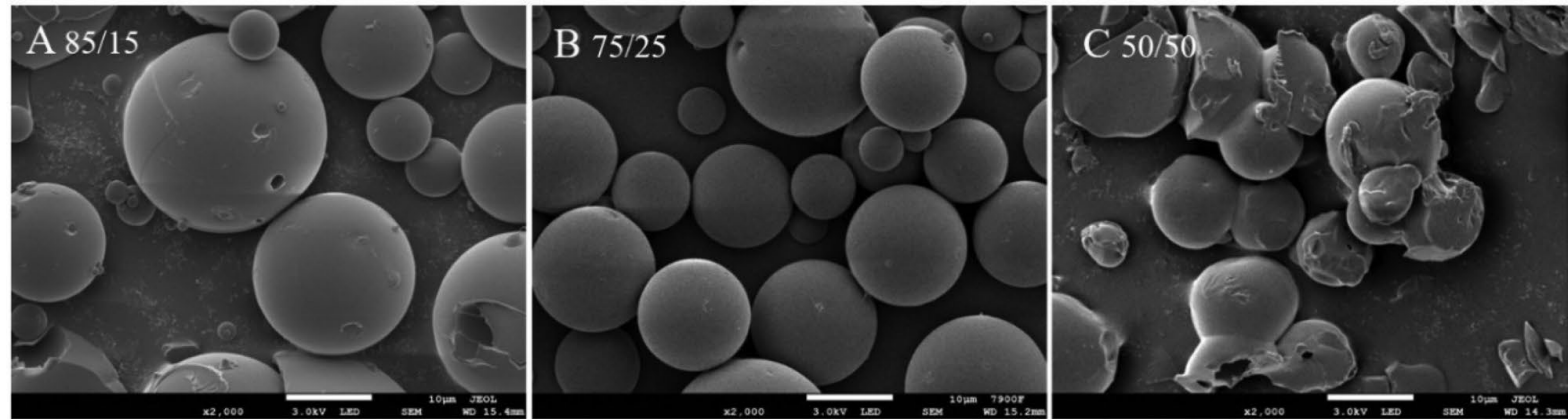
Effects of different PLGA models on microsphere morphology: (A) 85/15 PLGA; (B) 75/25 PLGA; (C) 50/50 PLGA.

When the LA: GA ratio of PLGA was 85:15, 75:25 and 50:50, the encapsulation efficiency of PLGA microspheres was 10.56%, 14.37% and 13.88%, respectively. The encapsulation efficiency of PLGA microspheres was at a low level, but PLGA 75:25 could improve the encapsulation efficiency. From Figure 4, with the change of LA/GA ratio in PLGA, the morphology of microspheres also showed different results, among which 75:25 was the best, 85:15 was the second, and 50:50 was the worst.

Based on the particle size, particle size distribution, encapsulation efficiency, and morphological results of the microspheres, PLGA7525 was finally selected as the best model for the preparation of GeXIVA[1,2] microspheres.

### 3.3. Optimization for preparing GeXIVA[1,2]-loaded PLGA microspheres with high EE

#### 3.3.1. PLGA concentration

Previous studies have reported that the PLGA concentration in O played a crucial role in EE, particle size and in vitro release behavior (Coll-Satue et al., 2021, El-Maghawry et al., 2020) (Table 3).

**Table 3.** The effect of PLGA concentration on the particle size and encapsulation efficiency of microspheres (*n* = 3).

PLGA concentration	Average particle size (μm)	Span	EE (%)
10 %	16.01 ± 0.11	1.03	14.37 ± 0.16
20 %	26.86 ± 0.11	0.94	50.55 ± 0.65
30 %	31.03 ± 0.37	1.29	56.08 ± 0.58

From the above results, it can be seen that the PLGA concentration in the oil phase increases by 30% from 10%, and the microspheres can maintain good morphology without obvious difference; the drug entrapment efficiency is significantly improved, and the average particle size becomes larger, but the distribution span of particle size is wider, which is consistent with the results in other studies.

**Figure 5.**
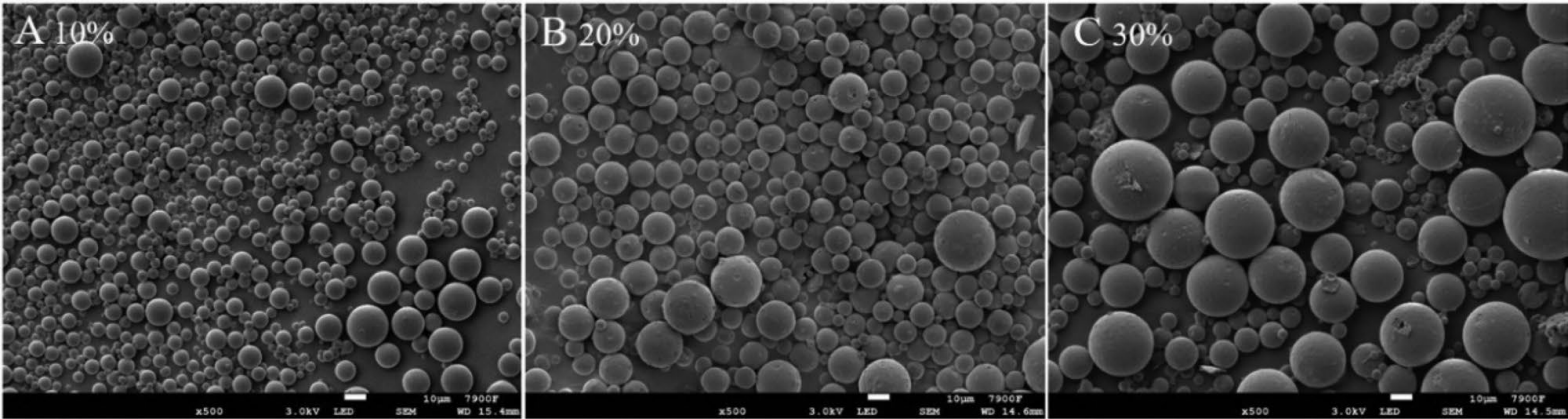
Effects of different PLGA concentrations on the morphology of microspheres: (A) 10%PLGA; (B) 20%PLGA; (C) 30%PLGA.

There are a variety of possible reasons: 1) the increase of PLGA concentration leads to the increase of oil phase viscosity, which prevents the drug from diffusing into the water phase; 2) the increase of oil phase viscosity may lead to the formation of larger emulsion droplets, which reduces the specific surface area and drug diffusion; 3) the high concentration of polymer accelerates the solidification of microspheres, which prevents the drug diffusion, and improves the drug loading and encapsulation efficiency for many reasons. However, with the increase of viscosity of oil phase, the dispersion of oil phase in water phase becomes uneven, which eventually leads to the increase of particle size distribution span of microspheres (Figure 5).

#### 3.3.2. PVA concentration

From Table 4, it can be seen that when the PVA concentration increases from 1.0% to 3.0%, the encapsulation efficiency and particle size of the microspheres decrease, but the distribution of particle size is more uniform. The possible reason is that with the increase of PVA concentration in the external water phase, the emulsion droplets formed in the emulsification process are smaller and the specific surface area increases, and GeXIVA[1,2] is easier to diffuse to the external water phase, so the encapsulation efficiency decreases.

**Table 4.** Effect of PVA concentration on particle size and encapsulation efficiency.

PVA concentration	Average particle size (μm)	Span	EE (%)
1.0%	31.03 ± 0.37	1.29	56.08 ± 0.58
2.0%	20.00 ± 0.13	1.15	50.66 ± 0.62
3.0%	13.82 ± 0.19	1.07	39.12 ± 0.49

#### 3.3.3. Concentration of NaCl in PVA solution

From the above results, it can be seen that the addition of NaCl to PVA is conducive to the improvement of entrapment efficiency. When the concentration of NaCl is 1.0%, the entrapment efficiency reaches the highest level of 72.57%. When the concentration is 2.0%, it is not helpful for the improvement of the entrapment efficiency but has a certain reducing effect. When 1.0% NaCl is added to the outer water phase, the osmotic pressure of the outer water phase is conducive to retaining GeXIVA[1,2] in the inner water phase, which is conducive to improving the encapsulation efficiency. When the concentration of NaCl in PVA is too high, the osmotic pressure of the outer water phase is large, which will cause the water in the inner water phase to migrate to the outer water phase, and at the same time lead to the leakage of GeXIVA[1,2], which will cause the encapsulation efficiency. There is a certain decline. When the concentration of NaCl is too high, a large number of pores will be formed on the surface of the microspheres, which will lead to the diffusion of GeXIVA[1,2] into the water when the microspheres solidify, and eventually reduce the entrapment efficiency. However, with the increase in NaCl concentration, the particle size and size distribution of the microspheres did not change (Table 5).

**Table 5:** Effect of NaCl concentration in PVA on particle size and encapsulation efficiency (*n* = 3).

Concentration of NaCl in PVA solution	Average particle size (μm)	Span	EE (%)
0%	31.03 ± 0.09	1.29	56.08 ± 0.58
0.5%	25.13 ± 0.12	1.20	65.18 ± 0.53
1.0%	21.55 ± 0.13	1.19	72.57 ± 0.46
2.0%	22.38 ± 0.06	1.15	70.56 ± 0.61

Generally speaking, when the concentration of NaCl in PVA is 1.0%, the encapsulation efficiency of the microspheres reaches the peak, and the particle size of the microspheres is about 20 μm, the span value is small, which meets the requirements of particle size and particle size uniformity. Therefore, it can be basically determined that the concentration of NaCl in the external aqueous phase is 1.0%.

#### 3.3.4. Types of protective agents added in inner water phase

According to the experimental results, the addition of protective agent in the internal water phase has no obvious effect on the particle size of microspheres. For the encapsulation rate, the encapsulation rate of microspheres can be improved to a certain extent, of which trehalose has the best effect, mannitol is the second, glycine is the worst, and even the encapsulation rate is slightly lower than that without protective agent (Table 6).

**Table 6:** Effect of inner water phase protective agent type on particle size and encapsulation efficiency (*n* = 3).

protective agents	Average particle size (μm)	Span	EE (%)
not added	21.55 ± 0.13	1.19	72.57 ± 0.46
Trehalose	23.11 ± 0.08	1.51	88.97 ± 0.73
Mannitol	23.31 ± 0.06	1.41	78.19 ± 0.71
Glycinate	21.15 ± 0.06	1.50	71.87 ± 0.67

The protective effect of trehalose on GeXIVA[1,2] may be due to the fact that trehalose has a certain viscosity, surrounded by GeXIVA[1,2], which can reduce the damage caused by GeXIVA[1,2] in oil water boundary to a certain extent and reduce the loss of GeXIVA[1,2]. Therefore, trehalose was added to the internal water phase as a protective agent to improve the encapsulation rate and stability of microspheres.

### 3.4 In vitro release of the GeXIVA[1,2] microspheres

**Figure 6.**
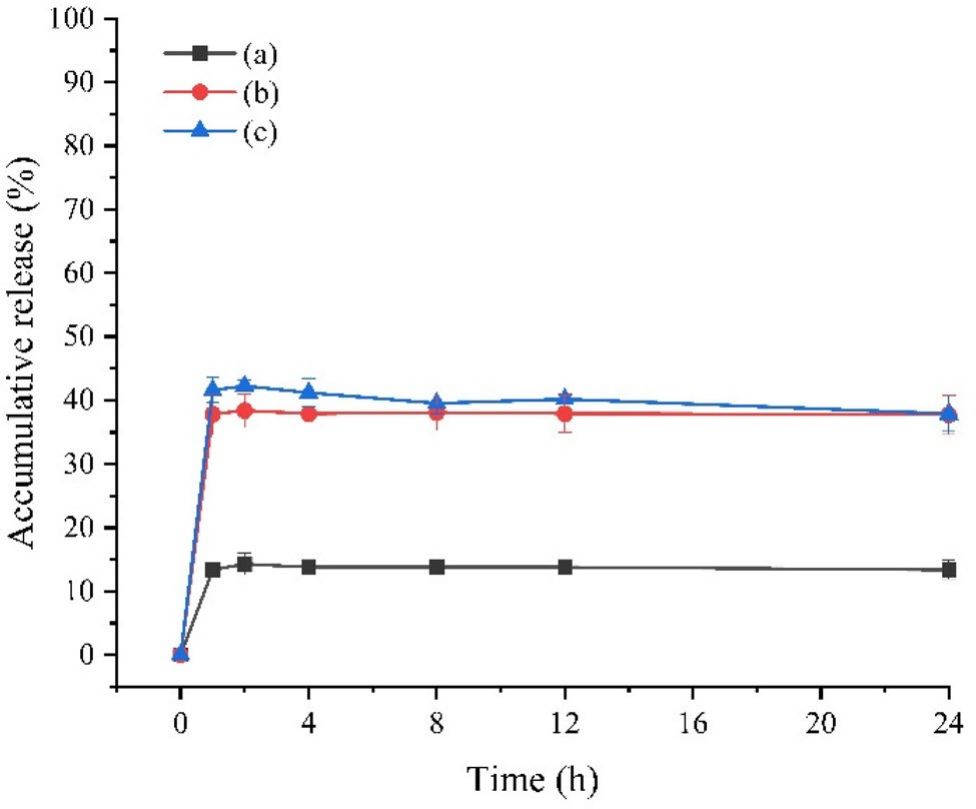
In vitro release of GeXIVA[1,2] microspheres for injection on the first day: (a) W1 without additives; (b) W1 adds 15% trehalose; (c) W1 adds 15% mannitol.

The sudden release of GeXIVA[1,2] microspheres is shown in Figure 6. The results showed that the burst release of microspheres was about 10% on the first day when the protective agent was not added in the microspheres. The burst release of microspheres was increased when the protective agent was added in the water phase of microspheres. The reason may be that GeXIVA[1,2] adsorbed on the surface of microspheres released rapidly, resulting in some sudden release. The addition of protective agent can increase the burst release rate. It may be that when trehalose or mannitol is added in the inner water phase, some additives in the inner water phase will be distributed at the oil-water interface. When the microspheres are solidified, these additives will stay in PLGA. When the microspheres are released, the additives will dissolve rapidly due to their good water solubility, forming certain pores in the microspheres carrier material, thus accelerating the release of some additives The sudden release of drugs.

**Figure 7.**
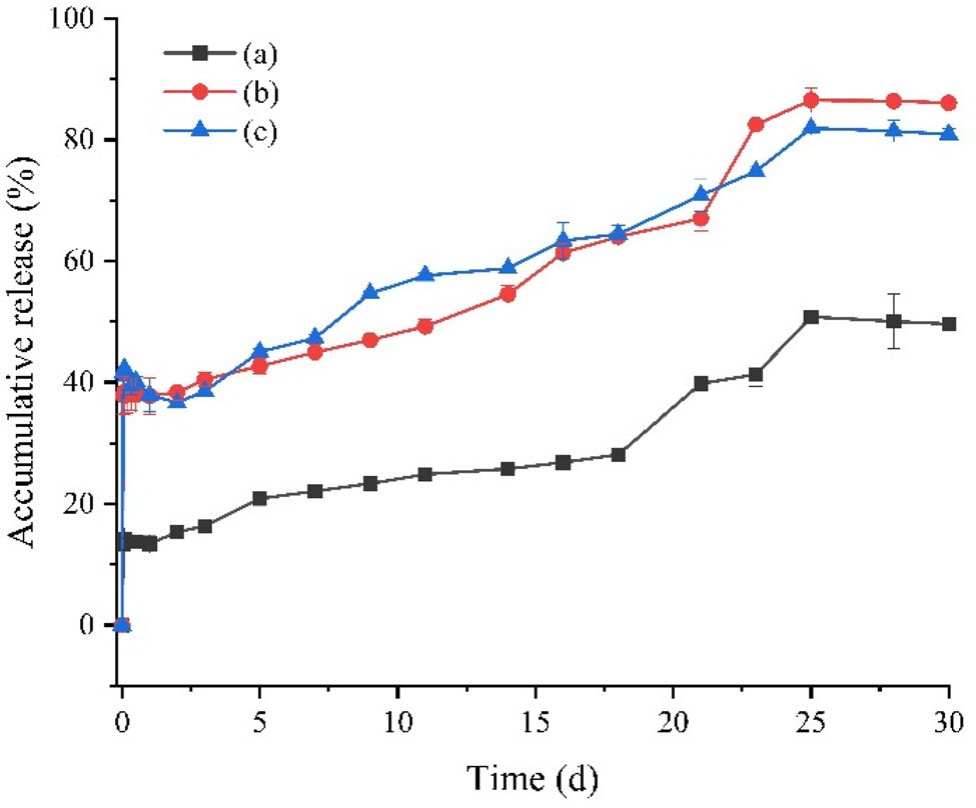
Cumulative in vitro release of GeXIVA[1,2] microspheres for injection over 30 days: (a) W1 without additives; (b) W1 adds 15% trehalose; (c) W1 adds 15% mannitol.

The cumulative release rate of the prescription without protective agent in the internal water phase was about 50% in 30 days, while the cumulative release rate of the prescription with protective agent in the internal water phase was more than 80%. The cumulative release rate of the prescription with trehalose in the internal water phase was 86.39%, and the cumulative release rate of the prescription with mannitol in the internal water phase was 81.43%. The results show that the addition of protective agent in the inner water phase can help to release GeXIVA[1,2] microspheres more completely (Figure 7).

The results of in vitro release showed that the burst release of microspheres mainly occurred in the first 4 hours, and then it could be released smoothly. For the target product, the first day of sudden release is about 37.73%, and the cumulative release rate is about 30 days, and the cumulative release rate is 86.39%. Due to the slow analgesic effect, the product can achieve rapid release within 4 hours. Therefore, for this product, the first day of sudden release is acceptable, and the sustained-release cycle of one month can significantly reduce the frequency of administration, reduce the possible adverse reactions, and improve the compliance of patients.

### 3.5. Protective mechanisms of trehalose on GeXIVA[1,2] in PLGA microspheres

#### 3.5.1. Differential scanning calorimetry (DSC)

**Figure 8.**
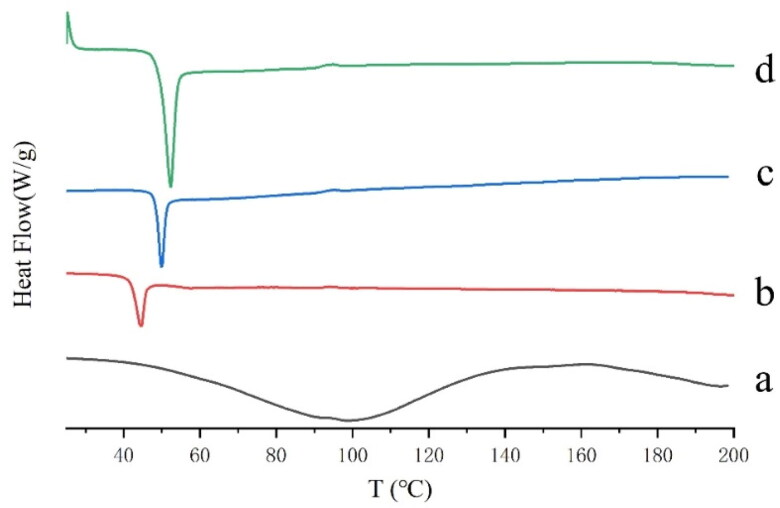
DSC patterns of GeXIVA[1,2] microsphere samples: (a) GeXIVA[1,2]; (b) PLGA; (c) GeXIVA[1,2] microspheres without protective agent in the inner water phase; (d) GeXIVA[1,2] microspheres with trehalose as protective agent in the inner water phase.

The pure GeXIVA[1,2] is amorphous, and PLGA is crystalline. Because the structure of GeXIVA[1,2] microspheres is PLGA to wrap GeXIVA[1,2] in the interior, PLGA materials still play a leading role in the thermal behavior of the samples after GeXIVA[1,2] preparation. Therefore, the melting temperature of the two GeXIVA[1,2] microspheres is only slightly increased compared with pure PLGA. However, from the small changes of the map, it can be seen that the melting peak position of the two GeXIVA[1,2] microspheres added with trehalose and the internal water without protective agent has a certain shift, indicating that the composition of the two microspheres is different, and the difference affects the thermal behavior of PLGA to a certain extent (Figure 8).

#### 3.5.2. Fourier transform infrared spectroscopy (FTIR)

**Figure 9.**
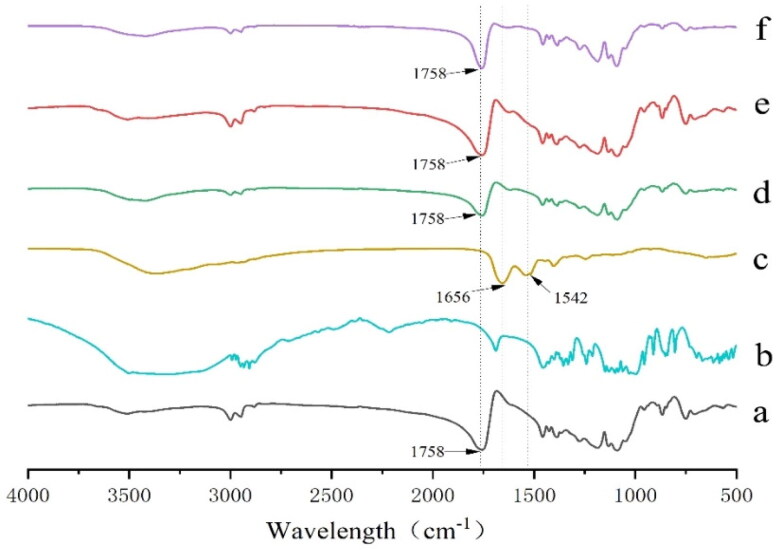
FT-IR spectra of GeXIVA[1,2] microsphere samples: (a) PLGA; (b) trehalose; (c) GeXIVA[1,2]; (d) physical mixing of GeXIVA[1,2], trehalose and PLGA; (e) GeXIVA without protective agent in the inner water phase [1,2] microspheres; (f) GeXIVA[1,2] microspheres with trehalose added as a protective agent in the inner water phase.

FTIR spectra showed that pure GeXIVA[1,2] showed two characteristic peaks at 1656 cm^−1^ and 1542 cm^−1^, which belonged to amide I band and amide II band of GeXIVA[1,2] respectively. As mentioned above, the structure of PLGA microspheres is usually the carrier material, PLGA encapsulates the drug inside, so GeXIVA[1,2] is encapsulated inside by PLGA. The spectral information of GeXIVA[1,2] is basically masked during Fourier transform infrared spectroscopy scanning, and the infrared spectral information of microspheres mainly comes from PLGA material. It can be seen from the results that the FT-IR spectrum information of GeXIVA[1,2] microspheres without adding protective agent in the inner water phase is basically consistent with that of pure PLGA, which is basically consistent with the prediction results. However, the FT-IR peak shape of GeXIVA[1,2] microspheres with trehalose as a protective agent in the inner aqueous phase is different from PLGA and GeXIVA[1,2] microspheres without protective agent in the wavelength range of 1700 cm^−1^ ∼ cm^−1^. The results showed that trehalose could interact with PLGA (Figure 9).

#### 3.5.3. Scanning electron microscope (SEM)

**Figure 10.**
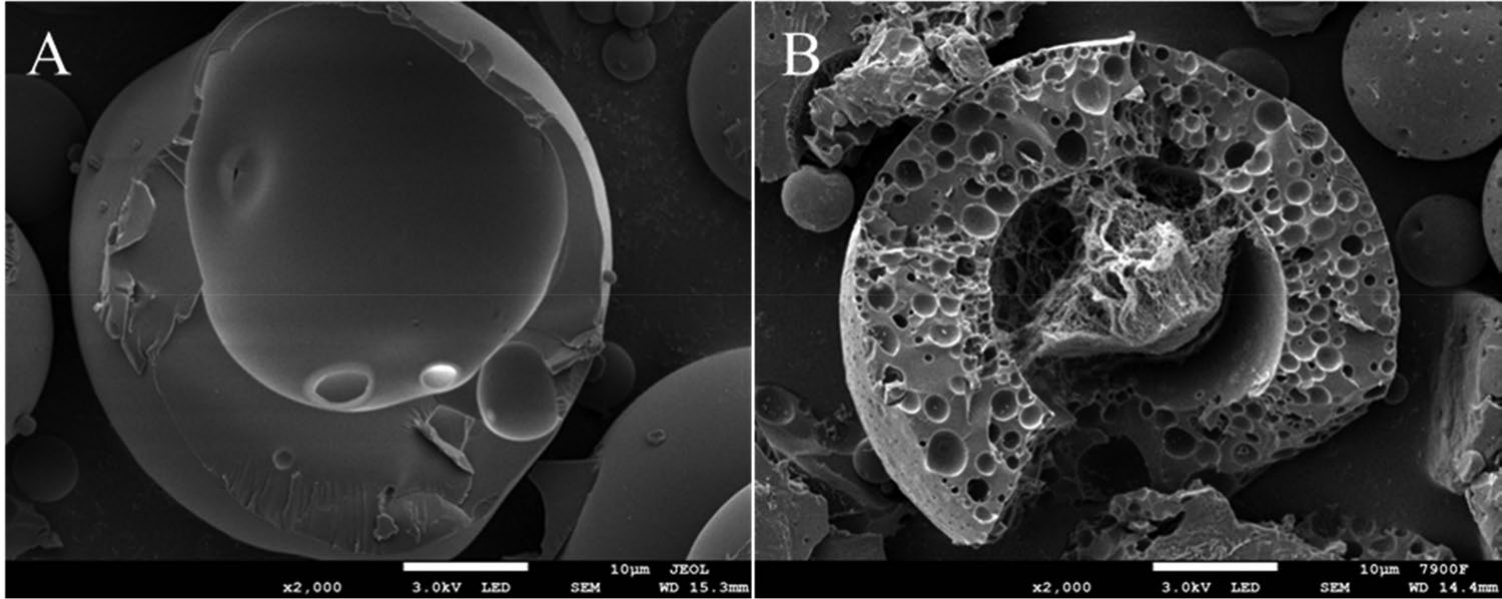
Internal structure of GeXIVA[1,2] microspheres.: (A) Trehalose is not added to the inner water phase; (B) Trehalose is added to the inner water phase.

From the results of scanning electron microscope (SEM) observation of the internal structure of the microspheres, it is obvious that the internal cavity of GeXIVA[1,2] microspheres without trehalose in the internal water phase is a cavity, and GeXIVA[1,2] can not be observed. The possible reason is that GeXIVA[1,2] is not successfully encapsulated or GeXIVA[1,2] is distributed in PLGA. However, when trehalose was added as a protective agent in the internal aqueous phase, it could be clearly observed that the lumpy contents were wrapped in the cavity formed by PLGA. This kind of structure is likely to be a glassy state formed by trehalose with high viscosity, which encapsulates GeXIVA[1,2] in trehalose, that is, trehalose ‘wears a layer of armor’ to GeXIVA[1,2] (Figure 10).

### 3.6. Molecular simulation

Molecular simulation aims to build a set of models and algorithms based on experi-ments or the basic principles, so as to calculate the reasonable molecular structure and molecular behavior (Van Gunsteren et al., 2018). It can simulate not only the static structure of molecules but also the dynamic behavior of molecular systems (Van Gunsteren et al., 2018). In this simulation, the molecular distributions of GeXIVA[1,2], trehalose and mannitol were characterized. The hydrogen bonds among GeXIVA[1,2] and excipients were predicted. The other interactions including van der Waals force and electrostatic interaction were calculated, too. Moreover, the secondary structure of GeXIVA[1,2] was concerned.

#### 3.6.1. Three-dimensional conformation

**Figure 11.**
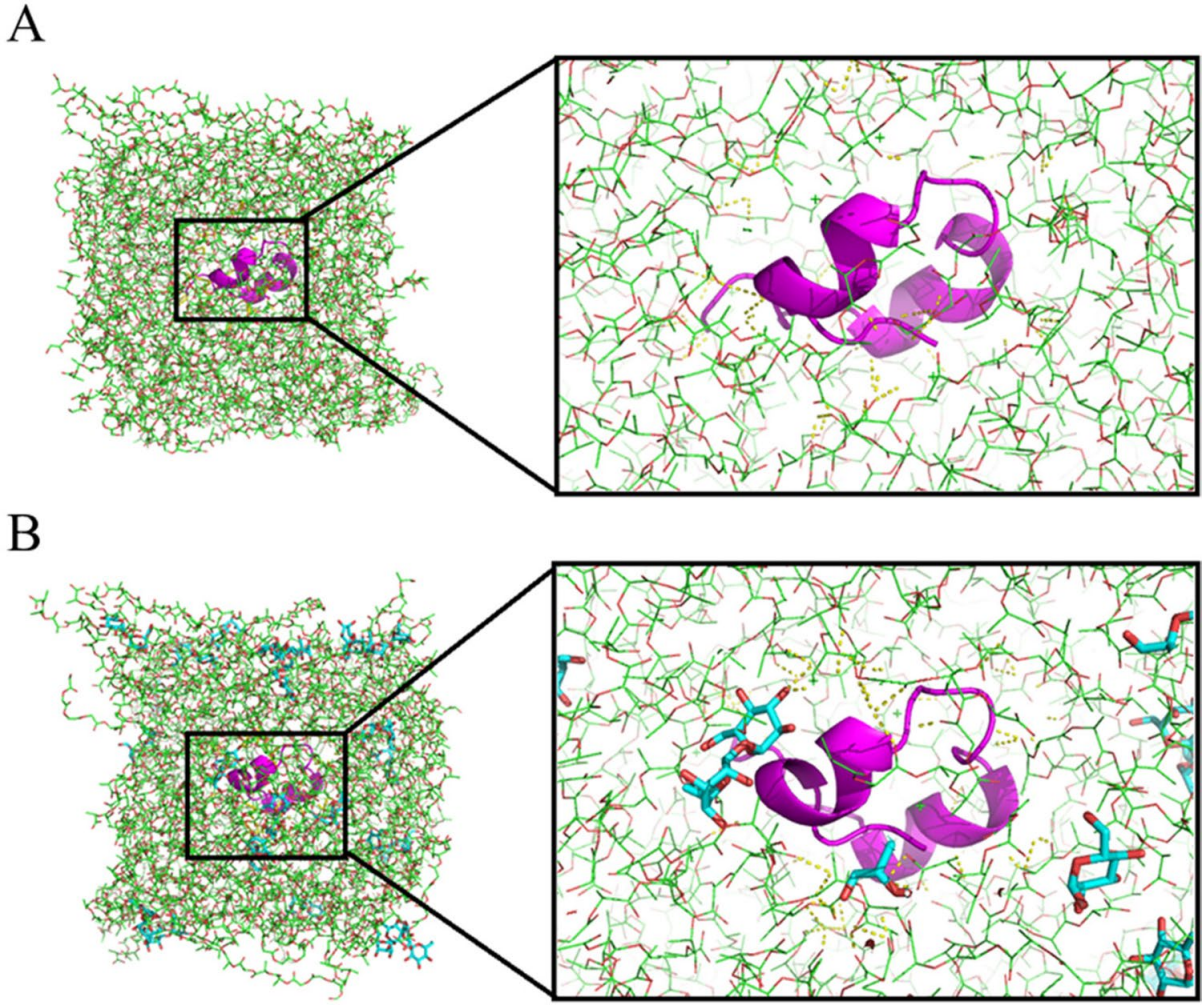
Three-dimensional conformation map of each molecule in the molecular simulation system: (A) GeXIVA [1,2]-PLGA system; (B) GeXIVA [1,2]-trehalose-PLGA system. GeXIVA[1,2] molecule (pink cartoon); sea bath sugar (light blue sticks); PLGA: (green lines); hydrogen bonds (yellow dots).

The distribution of molecules in the system can be obtained from the three-dimensional conformation. The results show that trehalose can be dispersed not only around GeXIVA[1,2], but also in PLGA. GeXIVA[1,2] can form hydrogen bond with trehalose and PLGA (Figure 11).

#### 3.6.2. Molecular distribution density and hydrogen bonding

**Figure 12.**
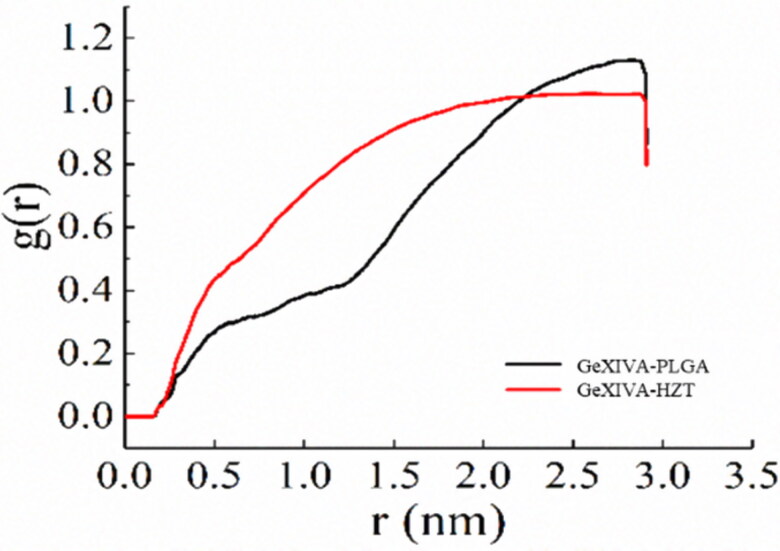
Distribution density of each molecule in GeXIVA[1,2]-trehalose-PLGA system.

The molecular distribution density of trehalose was higher than that of PLGA when the distance from GeXIVA[1,2] was less than 2.2 nm. When the distance is greater than 2.2 nm and less than 3.0 nm, the distribution density of PLGA is higher than trehalose. Thus, compared with PLGA, trehalose and GeXIVA[1,2] have more close interaction (Figure 12).

**Figure 13.**
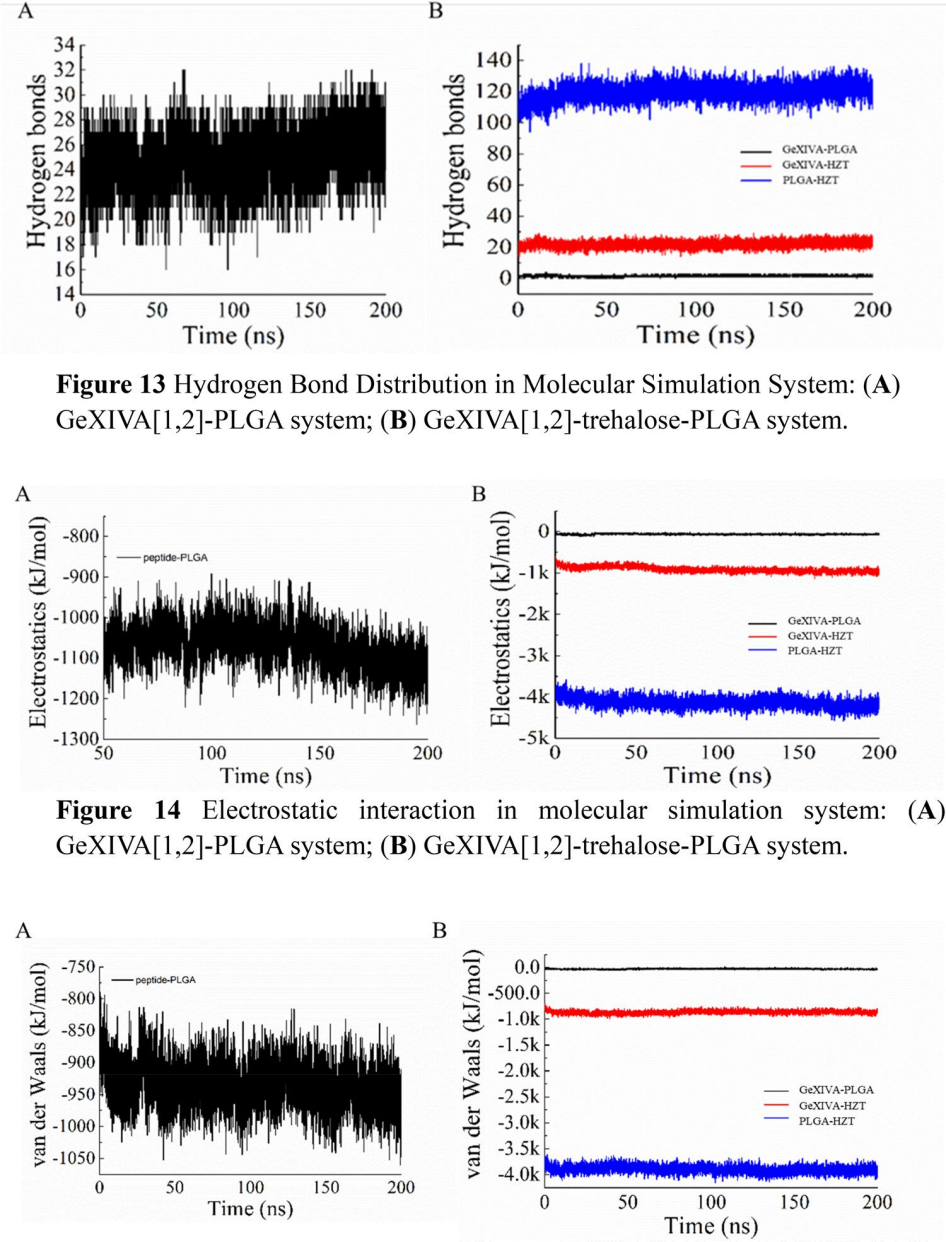
Hydrogen Bond Distribution in Molecular Simulation System: (A) GeXIVA[1,2]-PLGA system; (B) GeXIVA[1,2]-trehalose-PLGA system.

In the GeXIVA[1,2] - PLGA system, the number of hydrogen bonds formed between GeXIVA[1,2] and PLGA is about 20, while in the GeXIVA[1,2] - trehalose PLGA system, the number of hydrogen bonds formed between GeXIVA[1,2] and PLGA is significantly reduced, and the number of hydrogen bonds fluctuates from 1 to 3, while the number of hydrogen bonds formed between GeXIVA[1,2] and trehalose is about 20. The results showed that the hydrogen bond formed between GeXIVA[1,2] and PLGA was weakened by trehalose, but more hydrogen bond was formed between trehalose and GeXIVA[1,2]. In addition, more hydrogen bond was formed between trehalose and PLGA, which fluctuated in the range of 100 ∼ 125 (Figure 13).

#### 3.6.3. Electrostatic interactions and van der waals forces

**Figure 14.**
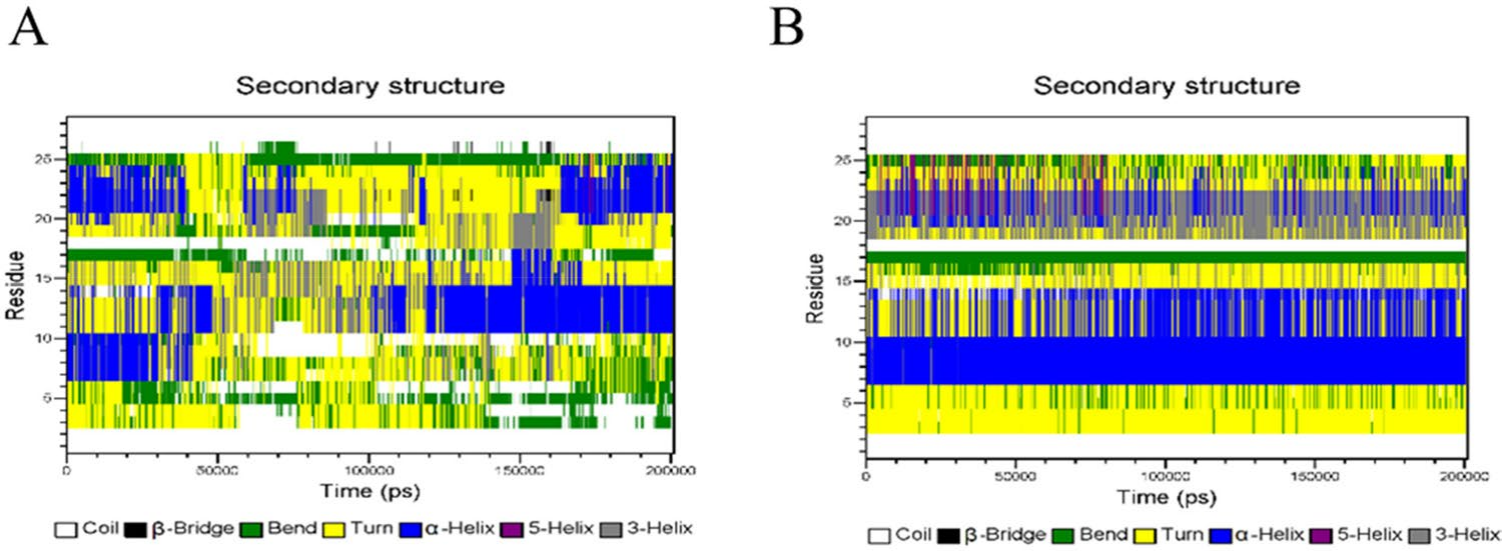
Electrostatic interaction in molecular simulation system: (A) GeXIVA[1,2]-PLGA system; (B) GeXIVA[1,2]-trehalose-PLGA system.

**Figure 15.**
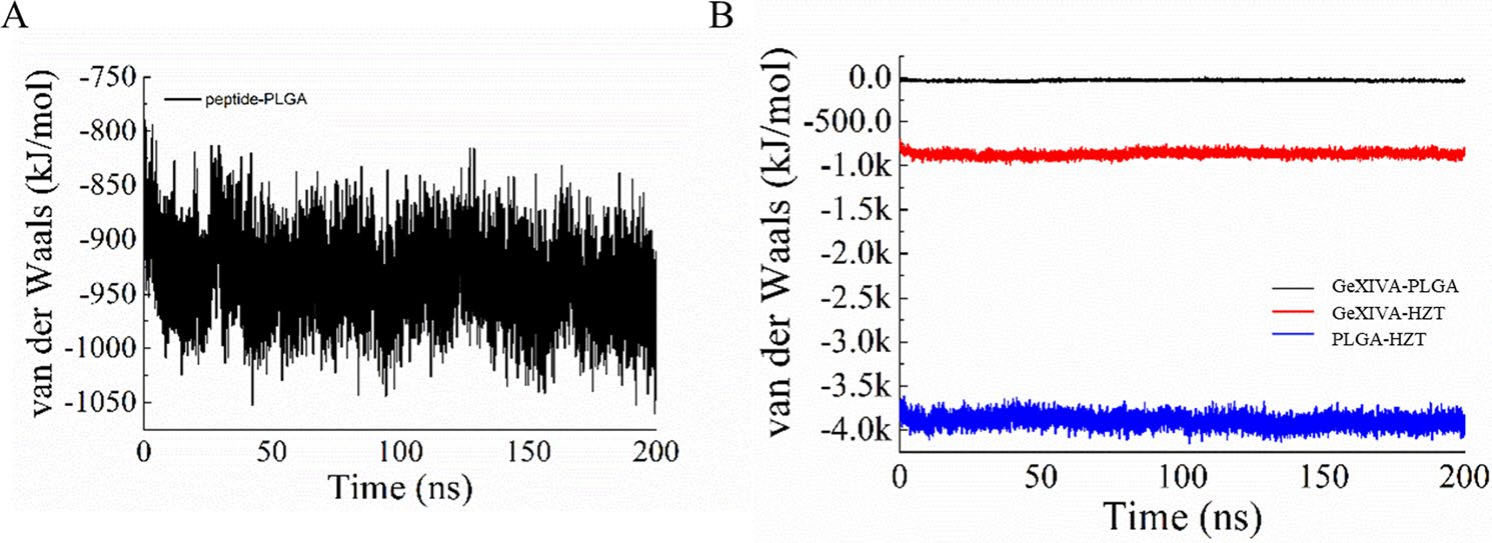
Van der Waals forces in molecular simulation systems: (A) GeXIVA[1,2]-PLGA system; (B) GeXIVA[1,2]-trehalose-PLGA system.

In GeXIVA[1,2] - PLGA system, the electrostatic force of GeXIVA[1,2] and PLGA fluctuates from − 900 to −1200 kJ/mol, and the van der Waals force fluctuates from −850 to −1000 kJ/mol. However, in GeXIVA[1,2] - trehalose PLGA system, the interaction between GeXIVA[1,2] and PLGA electrostatic force and van der Waals force is very weak, almost close to − 50 kJ/mol. However, the electrostatic force and van der Waals force of GeXIVA[1,2] and trehalose vary from − 1000 kJ/mol to − 1000 kJ/mol. The results showed that the electrostatic interaction and van der Waals force between GeXIVA[1,2] and PLGA were weakened by the involvement of trehalose. On the contrary, more binding force was produced between trehalose and GeXIVA[1,2]. At the same time, a stronger force was formed between trehalose and PLGA. The electrostatic force and van der Waals force were about − 4000 kJ/mol (Figures 14 and 15).

#### 3.6.4. Secondary structure of GeXIVA[1,2]

In the mixed system of GeXIVA[1,2] - PLGA, the secondary structure of GeXIVA[1,2] is unstable. The secondary structure of 6 ∼ 10 amino acid residues, 11 ∼ 14 amino acid residues and 20 ∼ 24 amino acid residues of GeXIVA[1,2] changes greatly in the simulation process. Specifically, for 6 ∼ 10 amino acid residues, the α - helix structure is maintained in the process of 0 ∼ 50 ns, but in the last 150 ns, the 6 ∼ 10 fragments will change to the state of turn curl; for 11 ∼ 14 amino acid residues, the α - helix structure will change from turn curl to α - helix; for 20 ∼ 24 amino acid residues, the α - helix structure will change to turn curl first, and finally stabilize in the α - helix structure.

**Figure 16.**
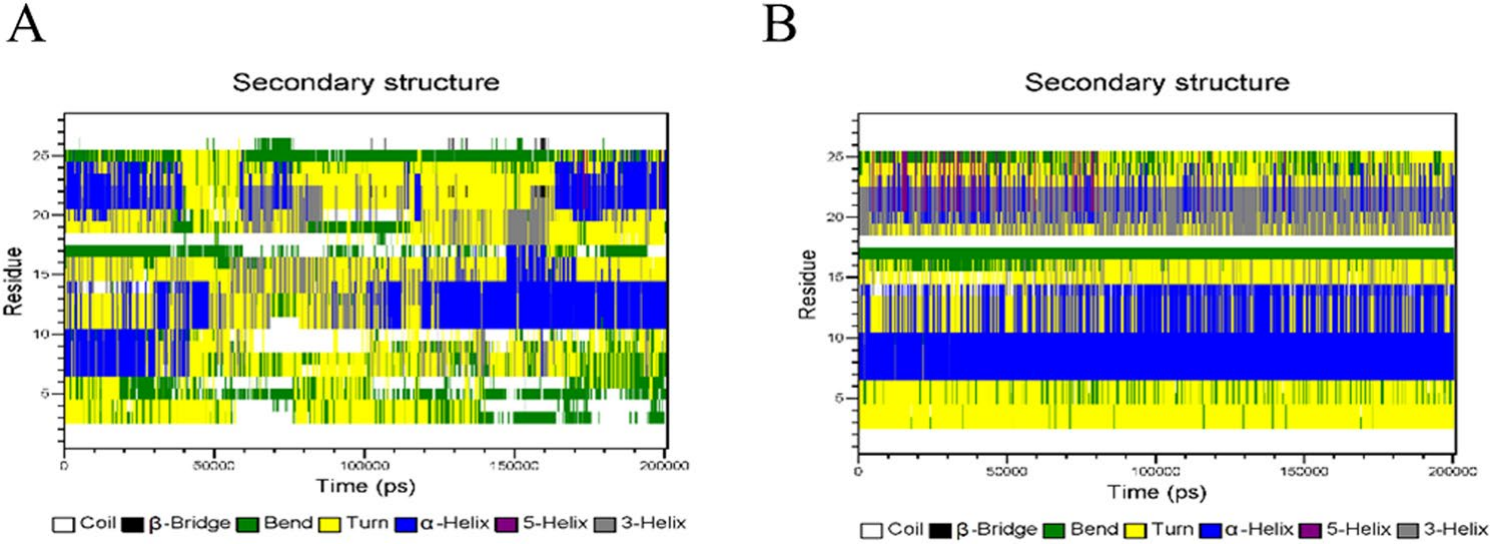
Secondary structure of GeXIVA[1,2] in a molecular simulation system: (A) GeXIVA[1,2]-PLGA system; (B) GeXIVA[1,2]-trehalose-PLGA system.

However, the secondary structure of GeXIVA[1,2] was more stable in the system of GeXIVA[1,2] - trehalose PLGA after adding trehalose in the internal aqueous phase, which was dominated by α - helix, turn coil and coil coil coil (Figure 16).

## 4. Conclusion

In this study, we investigated the effects of different formulation variables on the particle size and encapsulation efficiency of GeXIVA[1,2] microspheres. The particle size uniformity and encapsulation efficiency of the microspheres prepared based on the final formulation were significantly improved. The sustained-release cycle is 1 month, which can significantly reduce the frequency of administration, and improve patient compliance. In addition, we also explored the protective effect mechanism of trehalose against GeXIVA[1,2] in PLGA microspheres. The results of molecular simulation studies show that the secondary structure of GeXIVA[1,2] can be maintained and its stability can be improved with the participation of trehalose. Therefore, this study shows that GeXIVA[1,2] sustained-release microspheres are expected to be applied in the treatment of chronic neuropathic pain, which can achieve multiple coverage of neuropathic pain treatment, which has great clinical significance. This study also provides a certain reference for the protective mechanism of carbohydrates on proteins and peptides.
